# Editorial to “Left atrial anterolateral linear ablation for Biatrial tachycardia via Bachmann's bundle, interatrial septum, and left atrial Anterior Wall under mitral isthmus block”

**DOI:** 10.1002/joa3.12861

**Published:** 2023-05-13

**Authors:** Takeshi Kato

**Affiliations:** ^1^ Department of Cardiovascular Medicine Kanazawa University Graduate School of Medical Sciences Kanazawa Japan

Macroreentrant biatrial tachycardia is a rare form of macroreentrant atrial tachycardia reported first in 1998.^1^ The tachycardia is observed predominantly in patients with a history of open‐heart surgery, corrected congenital heart disease, or atrial fibrillation (AF) ablation. The circuit of biatrial tachycardia consists of two atria and two interatrial bridges which might serve as the critical isthmus.

The inter‐atrial conduction may occur over discrete muscular bands, most prominently the Bachmann's bundle. This structure is subepicardial and crosses the anterior interatrial groove from the medial base of the right atrial appendage to that of the left. Bachmann's bundle has always been found to be a broad structure, with median measurements of 4 mm in thickness × 9 mm in height and lower and upper lengths of 3 and 10 mm, respectively, overlying Waterson's groove.^2^ It is the primary mediator of synchronous biatrial activation. Muscle fibers in the walls of the coronary sinus connect the posteromedial right atrium (RA) with the posterior left atrium (LA) in ≈90% of patients. The true atrial septum comprises the fossa ovalis, and conduction via this structure has been demonstrated in ≈30% of adult patients. Moreover, ≈70% of healthy adults have conduction over small muscular bridges between the posterior LA near the right pulmonary vein ostia and the intercaval posterior RA. Although this inter‐atrial conduction could be a potential target for catheter ablation of biatrial tachycardia, inter‐atrial connections are anatomically robust, with complex insertions over a wide area and epicardial connections. This may make the interatrial connections an unattractive target for the ablation of biatrial tachycardia and ablation at a critical isthmus distant to the interatrial connection may have greater success.^3^ Another concern for biatrial tachycardia ablation is that it could easily cause severe atrial conduction disturbance, electrical isolation of the left atrial appendage, and an atrio‐ventricular block.

In this issue of the *Journal of Arrhythmia*,^4^ Sekihara et al reported an interesting case of biatrial tachycardia after extensive ablation of LA for atrial fibrillation. The ultra‐high‐density mapping revealed counter‐clockwise biatrial tachycardia via Bachmann's bundle, interatrial septum, and LA anterior wall. The SKYLINE™ software presented an interval of 20 ms during which both atria were electrically silent, suggesting the role of epicardial Bachmann's bundle as a critical isthmus of the tachycardia circuit. The tachycardia was terminated by the radiofrequency application at the LA anterior wall, which was probably a part LA junction of Bachmann's bundle. However, the inter‐atrial conduction via Bachmann's bundle was not completely eliminated by this ablation and the atrial tachycardia recurred. In the following session, they created a new linear lesion lateral to the previous one to ‘isolate’ the BB breakthrough site from LA and successfully confirmed the bidirectional BB conduction block without compromising residual atrial conduction. Sekihara et al are to be congratulated for proposing a novel option for biatrial tachycardia ablation.

Finally, with the development and spread of ultra‐high‐density 3D mapping systems, we now have extensive knowledge of biatrial tachycardia. However, the treatment of biatrial tachycardia is still complicated and challenging to date. The most important thing to keep in our mind is to avoid groundless extensive ablation for atrial fibrillation which would produce troublesome biatrial tachycardia.^5^ We all know that “Prevention is Better than Cure.”

## CONFLICT OF INTEREST STATEMENT

Authors declare no Conflict of Interests for this article.
